# The potential protective role of peripheral immunophenotypes in Alzheimer’s disease: a Mendelian randomization study

**DOI:** 10.3389/fnagi.2024.1403077

**Published:** 2024-06-05

**Authors:** Chun-yan Zuo, Zhengwei Hu, Xiao-yan Hao, Meng-jie Li, Jing-jing Shi, Meng-nan Guo, Dong-rui Ma, Shuang-jie Li, Yuan-yuan Liang, Chan Zhang, Cheng-yuan Mao, Yuming Xu, Chang-he Shi

**Affiliations:** ^1^Department of Neurology, The First Affiliated Hospital of Zhengzhou University, Zhengzhou University, Zhengzhou, Henan, China; ^2^Academy of Medical Sciences of Zhengzhou University, Zhengzhou, Henan, China; ^3^NHC Key Laboratory of Prevention and Treatment of Cerebrovascular Diseases, The First Affiliated Hospital of Zhengzhou University, Zhengzhou University, Zhengzhou, Henan, China; ^4^Henan Key Laboratory of Cerebrovascular Diseases, The First Affiliated Hospital of Zhengzhou University, Zhengzhou University, Zhengzhou, Henan, China; ^5^Institute of Neuroscience, Zhengzhou University, Zhengzhou, Henan, China

**Keywords:** peripheral immunity, peripheral blood immune cell phenotype, Alzheimer’s disease, Mendelian randomization, protective factor

## Abstract

**Introduction:**

Alzheimer’s disease (AD) is the most widespread neurodegenerative disease in the world. Previous studies have shown that peripheral immune dysregulation plays a paramount role in AD, but whether there is a protective causal relationship between peripheral immunophenotypes and AD risk remains ambiguous.

**Methods:**

Two-sample Mendelian randomization (MR) was performed using large genome-wide association study (GWAS) genetic data to assess causal effects between peripheral immunophenotypes and AD risk. Utilizing the genetic associations of 731 immune cell traits as exposures. We adopted the inverse variance weighted method as the primary approach. The Weighted median and MR-Egger regression methods were employed as supplements. Various sensitivity analyses were performed to assess the robustness of the outcomes.

**Results:**

Based on the IVW method, we identified 14 immune cell traits that significantly reduced the risk of AD, of which six demonstrated statistical significance in both IVW and Weighted median methods. Among the seven immune traits, four were related to regulatory T (Treg) cells : (1) CD25++ CD45RA- CD4 not regulatory T cell % T cell (odds ratio (OR) [95% confidence interval (CI)] = 0.96 [0.95, 0.98], adjusted *P* = 1.17E−02), (2) CD25++ CD45RA- CD4 not regulatory T cell % CD4+ T cell (OR [95% CI] = 0.97 [0.96, 0.99], adjusted *P* = 3.77E−02), (3) Secreting CD4 regulatory T cell % CD4 regulatory T cell (OR [95% CI] = 0.98 [0.97, 0.99], adjusted *P* = 7.10E−03), (4) Activated & secreting CD4 regulatory T cell % CD4 regulatory T cell(OR [95% CI] = 0.98 [0.97, 0.99], adjusted *P* = 7.10E−03). In addition, HLA DR++ monocyte % monocyte (OR [95% CI] = 0.93 [0.89, 0.98], adjusted *P* = 4.87E−02) was associated with monocytes, and HLA DR on myeloid Dendritic Cell (OR [95% CI] = 0.93 [0.89, 0.97], adjusted *P* = 1.17E−02) was related to dendritic cells (DCs).

**Conclusion:**

These findings enhance the comprehension of the protective role of peripheral immunity in AD and provide further support for Treg and monocyte as potential targets for immunotherapy in AD.

## 1 Introduction

Alzheimer’s disease (AD) is comprised of a group of primary neurodegenerative diseases with unspecified etiology, representing the most common type of dementia. The most characteristic pathological changes in AD are the accumulation of neuroinflammatory extracellular β-amyloid (Aβ) deposits and intracellular hyperphosphorylated tau protein neurofibrillary tangles (NFT) (Serrano-Pozo et al., 2011; Reitz and Mayeux, 2014). Neuroinflammation and immune system dysregulation are prominent drivers of the development of AD. Regarding neuroinflammation, microglia and their phagocytic capacity are the focus of attention in the present work (Hemonnot et al., 2019). Studies have shown that microglia surrounding Aβ plaques in the central nervous system (CNS) activate and eliminate plaques and reduce their accumulation (Hansen et al., 2018). However, immunotherapeutic agents targeting the reduction of neuroinflammatory extracellular Aβ and tau proteins have failed in several clinical and animal trials in recent years, accompanied by serious immune-related side effects, suggesting a deficiency in the recognition of the immune mechanisms of AD (Wilcock et al., 2004; Sperling et al., 2012; Sevigny et al., 2016).

Previous studies have revealed that the blood-brain barrier is compromised before the onset of AD and that the central nervous system is not “immune privileged,” providing the possibility for the brain and peripheral immune cells to inter-communicate (Carson et al., 2006; Desai et al., 2007). In this neuroinflammatory response, the upregulation of the cell adhesion molecule (CAM) and CAM ligand expression on blood-brain–barrier endothelial cells mediate peripheral immune cells, prompting them to cross the blood-brain barrier and interact with immune cells residing in the CNS (Engelhardt and Ransohoff, 2005). The subpopulations of peripheral blood immune cell types are sophisticated and diverse, and each subpopulation and its cytokines exert various or opposing influences on AD development (Skias et al., 1985; Ziegler-Heitbrock, 2007; Lueg et al., 2015; Xu and Jia, 2021; Aries and Hensley-Mcbain, 2023). The intricate relationship between the peripheral immune system and AD can be adequately understood only by systematic and exhaustive studies of different subgroups.

Mendelian randomization is a robust type of analysis capable of dodging confounding and reverse causality bias by using genetic variation associated with exposure as an instrumental variable (IV) or a proxy instrumental variable to evaluate the causal effects of exposure on outcome (Davey Smith and Ebrahim, 2003; Burgess et al., 2012). The assignment of genetic variation is random and not influenced by environment or lifestyle during gametogenesis during pregnancy. Consequently, compared with traditional observational studies, Mendelian randomization studies can avoid confounding factors and the bias of reverse causality (Lawlor et al., 2008).

Previous studies on peripheral blood immune cell subpopulations in AD have been mainly observational, based on the number of the proportional changes of the subpopulations observed in AD patients or animal models. In contrast, relatively few studies have directly investigated the correlation between the traits of various peripheral blood immune cells and AD. With a comprehensive genome-wide association study (GWAS) dataset of peripheral blood immune cell phenotypes now available, MR provides a robust analytical approach to further explore the interplay of peripheral blood immune cell biomarkers in AD risk, which is rarely conducted in this field. We hypothesized that the subtypes in peripheral immunophenotypes have a proximate causal effect on AD risk. To determine this relationship, we performed a two-sample MR analysis using the GWAS data of the largest peripheral immunophenotypes as exposures to further explore their causal roles in AD risk.

## 2 Materials and methods

### 2.1 Data sources

This study strictly adhered to the STROBE-MR guidelines (Skrivankova et al., 2021). We employed a two-sample MR approach to investigate the relationship between peripheral blood immune cell traits and AD risk. GWAS data for peripheral immune cell traits, including 731 immune traits, were collected from 3,757 general populations from the east-central coast of Sardinia, Italy, in a population-based prospective study (Orrù et al., 2020). GWAS summary data for outcomes were obtained from the European Alzheimer’s and Dementia Biobank (EADB), including 85,934 cases (39,106 clinically diagnosed cases, 46,828 proxy cases) and 401,577 controls (Bellenguez et al., 2022). Additionally, use GWAS summary data from the International Genomics of Alzheimer’s Project (IGAP) as a validation cohort, comprising 17,008 individuals with an AD diagnosis and 37,154 healthy individuals (Lambert et al., 2013).

### 2.2 Instrument selection

**FIGURE 1:**
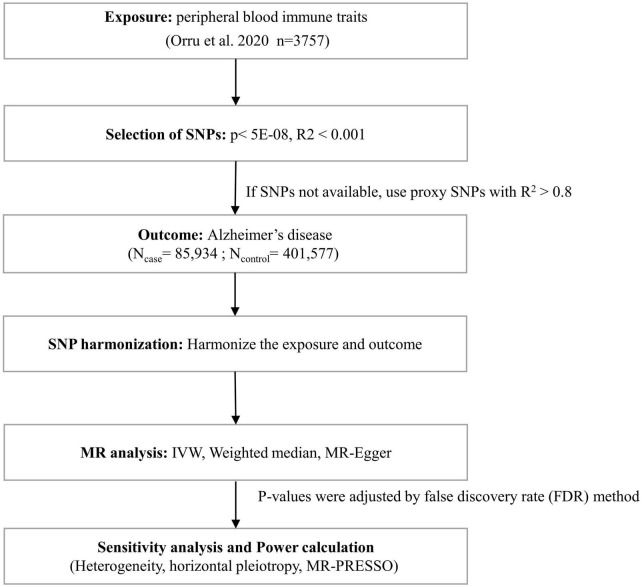
Flow chart for statistical analysis. Flow chart for the Mendelian randomization analysis exploring effects of peripheral blood immune traits on Alzheimer’s disease. SNP, single nucleotide polymorphism; IVW, inverse variance weighted; MR-PRESSO, Mendelian Randomization Pleiotropy RESidual Sum and Outlier.

Three basic assumptions are required to screen unbiased and eligible instrumental variables in MR studies exploring the association between peripheral blood immune cell traits and AD risk as follows (Glymour et al., 2012; Hemani et al., 2018): (1) correlation hypothesis: genetic variants as instrumental variables are intimately associated with risk factors of interest; (2) independence hypothesis: genetic variants are not associated with any confounding factors affecting exposure-outcome associations; and (3) exclusion hypothesis: genetic variants affect outcomes exclusively through risk factors. Accordingly, in compliance with the above assumptions, we performed the following filtering measures (Figure 1). First, the correlation hypothesis of MR was confirmed by screening single nucleotide polymorphisms (SNPs) that were significantly associated with peripheral immune cell traits from the GWAS summary data using *p* < 5E−08. Second, to ensure that the genetic variants of the IVs used were independent, we clustered the extracted SNPs based on the 1000 Genomes Project linkage disequilibrium (LD) structure. We clumped SNPs (R^2^ < 0.001 with any other associated SNP within 1,000 kb) and retained the SNPs with the lowest P-values. Third, we extracted these instrumental variables from the GWAS AD summary data. If no SNPs in the GWAS summary data of AD satisfied the above criteria, proxy SNPs strongly correlated with exposure (R^2^ > 0.8) were selected. Fourth, we harmonized the dataset to align the effect alleles for exposure and outcome (Hartwig et al., 2016). Additionally, we calculated F-statistics for each exposure to quantify the strength of the instrumental variables and then elected those traits with F-statistics > 10 (Burgess and Thompson, 2011; Pierce et al., 2011).

### 2.3 Power calculation

Power analysis of each exposure was performed using a web tool,^1^ with a Type-I error rate *a* = 0.05 and the estimated OR from the IVW method (Brion et al., 2013; Burgess, 2014).

### 2.4 Sensitivity analyses

Inverse variance weighting (IVW) was chosen as the main method of two-sample MR analysis to explore the causal relationship between exposure and outcome (Woolf et al., 2022). The Weighted median (Bowden et al., 2016) and MR-Egger regression methods (Bowden et al., 2015) were complementary. Subsequently, we performed a sensitivity analysis to examine the robustness of the results. The MR-Egger intercept determined whether the results had horizontal multiplicity, indicating the presence of horizontal multiplicity when the intercept significantly deviates from zero. We used Cochran’s Q statistic (Bowden et al., 2019) to test for heterogeneity in IV, with a *Q*-value > 0.05 indicating no heterogeneity among the instrumental variables. In addition, we used the MR pleiotropy residual sum and outlier (MR-PRESSO) method to detect horizontal pleiotropy (MR-PRESSO global test) (Verbanck et al., 2018). If horizontal pleiotropy was detected, horizontal pleiotropy was corrected using the MR-PRESSO outlier test to obtain unbiased causal estimates. For exposures with no more than three instrumental SNPs, pleiotropy analyses were performed using the PhenoScanner database, querying for other relevant traits found in previously published GWAS data that influenced the outcome and removing these SNPs to obtain a robust analysis (Kamat et al., 2019). Leave-one-out analysis (LOO) was performed to detect the presence of outliers substantially affected the causal effect. The odds ratio (OR) was applied to represent causality, for AD is a binary outcome (Palmer et al., 2011). Since exposure (peripheral immune cell traits) was repeatedly compared with the outcome (AD), *P*-values were corrected via the false discovery rate (FDR) method. All MR analyses were performed via R software (v.4.1.3). Two-sample MR analyses were performed using the TwoSampleMR package (v.0.5.6) and MRPRESSO (v.1.0) (Hemani et al., 2018; Verbanck et al., 2018).

## 3 Results

### 3.1 Overview

**FIGURE 2:**
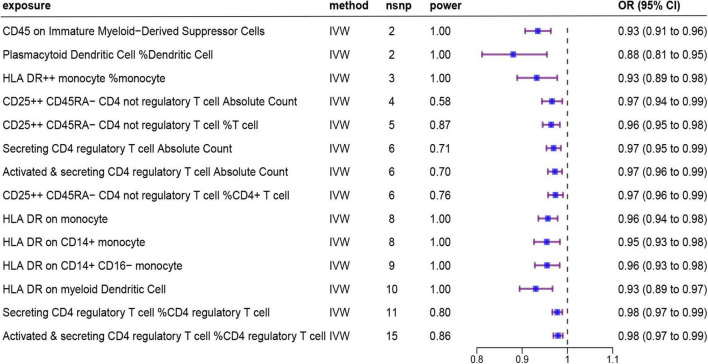
Forest plot showing results from the IVW method to evaluate potential causal associations between 14 protective immune cell traits and Alzheimer’s disease. IVW, inverse variance weighted; nsnp, the number of single nucleotide polymorphism; OR, odds ratio; CI, confidence interval.

Details of the 731 immune traits in the peripheral blood analyzed in this study are provided in Supplementary Table 1. The results of the MR analysis of these immune cell traits are shown in Supplementary Tables 2–5. Based on IVW as the primary analysis method for MR, after FDR correction, we identified 14 remarkable results with protective effects on AD, as shown in Figure 2 and Table 1, of which six traits showed significant negative relationships in both the IVW method and the weighted median method. No proxy SNPs were used in our analysis. The minimum value of the calculated F-statistic for the instrumental variables was above 60, indicating that all selected IVs represent robust instrumental variables. Additionally, among the six protective immune traits mentioned, we selected specific SNPs based on a threshold of *p* < 5E−08, LD window of 10,000 kb, and R^2^ = 0.001, which map to a total of eight genes encoding proteins (Supplementary Table 9). Among them, FCGR3A, MICB, IL2RA, and NEK7 encode proteins involved in immune regulation and inflammation, suggesting their potential as modulatory genes.

**TABLE 1:** Summary of the causal relationships of 14 protective immune cell traits on Alzheimer’s disease (AD) with various Mendelian randomization (MR) methods.

Exposure	SNP N	IVW		Weighted median		MR-Egger		F statistic
		OR (95%CI)	*P*-value (adjusted)	OR (95%CI)	P-value (adjusted)	OR (95%CI)	*P*-value (adjusted)	
CD25++ CD45RA- CD4 not regulatory T cell % T cell	5	0.96 (0.95, 0.98)	1.17E−02	0.96 (0.94, 0.98)	2.40E−02	0.99 (0.93, 1.05)	0.96	61.76
CD25++ CD45RA- CD4 not regulatory T cell Absolute Count	4	0.97 (0.94, 0.99)	4.76E−02	0.96 (0.94, 0.99)	1.02E−01	0.99 (0.93, 1.04)	0.95	66.78
Secreting CD4 regulatory T cell Absolute Count	6	0.97 (0.95, 0.99)	1.13E−02	0.97 (0.95, 0.99)	7.13E−02	0.99 (0.96, 1.02)	0.95	61.34
Activated & secreting CD4 regulatory T cell Absolute Count	6	0.97 (0.96, 0.99)	1.68E−02	0.98 (0.96, 1.00)	1.09E−01	0.99 (0.96, 1.02)	0.95	60.37
CD25++ CD45RA- CD4 not regulatory T cell % CD4+ T cell	6	0.97 (0.96, 0.99)	3.77E−02	0.97 (0.94, 0.99)	2.40E−02	1.00 (0.96, 1.05)	0.96	71.30
Secreting CD4 regulatory T cell % CD4 regulatory T cell	11	0.98 (0.97, 0.99)	7.10E−03	0.98 (0.96, 0.99)	2.40E−02	0.98 (0.96, 1.00)	0.53	110.27
Activated & secreting CD4 regulatory T cell % CD4 regulatory T cell	15	0.98 (0.97, 0.99)	7.10E−03	0.98 (0.96, 0.99)	2.40E−02	0.98 (0.96, 0.99)	0.53	107.06
HLA DR++ monocyte % monocyte	3	0.93 (0.89, 0.98)	4.87E−02	0.92 (0.88, 0.97)	2.40E−02	0.94 (0.68, 1.31)	0.96	118.81
HLA DR on CD14+ CD16- monocyte	9	0.96 (0.93, 0.98)	3.70E−02	0.96 (0.93, 1.00)	2.02E−01	1.04 (0.90, 1.19)	0.95	343.53
HLA DR on CD14+ monocyte	8	0.95 (0.93, 0.98)	3.77E−02	0.96 (0.93, 1.00)	2.22E−01	1.04 (0.89, 1.20)	0.95	318.91
HLA DR on monocyte	8	0.96 (0.94, 0.98)	7.10E−03	0.97 (0.94, 0.99)	9.78E−02	0.99 (0.93, 1.04)	0.95	251.56
Plasmacytoid Dendritic Cell %Dendritic Cell	2	0.88 (0.81, 0.95)	4.00E−02	NA	NA	NA	NA	168.98
CD45 on Immature Myeloid-Derived Suppressor Cells	2	0.93 (0.91, 0.96)	5.17E−03	NA	NA	NA	NA	166.65
HLA DR on myeloid Dendritic Cell	10	0.93 (0.89, 0.97)	1.17E−02	0.92 (0.89, 0.95)	8.61E−04	0.84 (0.76, 0.93)	0.53	394.40

AD, Alzheimer’s disease; MR, Mendelian randomization; IVW, inverse variance weighted; SNP, single nucleotide polymorphism; OR, odds ratio; CI, confidence interval; NA, not applicable.

### 3.2 Regulatory T cell-related protective traits and AD

In the current study, we identified seven regulatory T (Treg) cell–associated immune traits that are protective against AD based on the IVW method. The association statistics are summarized in Table 1. The results demonstrated that after FDR correction, the following four phenotypes associated with Treg cells showed remarkable protective effects against AD in both IVW analysis and Weighted median analysis (Supplementary Figure 1): (1) CD25++ CD45RA- CD4 not regulatory T cell % T cell in IVW analysis method (OR [95% confidence interval (CI)] = 0.96 [0.95, 0.98], adjusted *P* = 1.17E−02) and Weighted median analysis (OR [95% CI] = 0.96 [0.94, 0.98], adjusted *P* = 2.40E−02); (2) CD25++ CD45RA- CD4 not regulatory T cell % CD4+ T cell in IVW analysis method (OR [95% CI] = 0.97 [0.96, 0.99], adjusted *P* = 3.77E−02) and Weighted median analysis (OR [95% CI] = 0.97 [0.94, 0.99], adjusted *P* = 2.40E−02); (3) Secreting CD4 regulatory T cell % CD4 regulatory T cell in IVW analysis method (OR [95% CI] = 0.98 [0.97, 0.99], adjusted *P* = 7.10E−03) and Weighted median analysis (OR [95% CI] = 0.98 [0.96, 0.99], adjusted *P* = 2.40E−02); and (4) Activated & secreting CD4 regulatory T cell % CD4 regulatory T cell in IVW analysis method (OR [95% CI] = 0.98 [0.97, 0.99], adjusted *P* = 7.10E−03) and Weighted median analysis (OR [95% CI] = 0.98 [0.96, 0.99], adjusted *P* = 2.40E−02). In addition, MR-Egger results tended to be in the same direction, excepting CD25++ CD45RA- CD4 not regulatory T cell % CD4+ T cell. The remaining three Treg cell-associated immunophenotypes (Secreting CD4 regulatory T cell Absolute Count, Activated & secreting CD4 regulatory T cell Absolute Count and CD25++ CD45RA- CD4 not regulatory T cell Absolute Count) were only shown to reduce AD risk by IVW analysis (Supplementary Figure 1). However, the weighted median analysis and MR-Egger results trended in the same direction.

Subsequently, extensive sensitivity analyses verified the causal relationship between Treg cell-related immunophenotypes (CD25++ CD45RA- CD4 not regulatory T cell % T cell, CD25++ CD45RA- CD4 not regulatory T cell % CD4+ T cell, Secreting CD4 regulatory T cell Absolute Count and Activated & secreting CD4 regulatory T cell Absolute Count) and AD. Sensitivity analysis based on Cochran’s Q test (*Q*-value > 0.05) and MR-Egger intercept (MR-Egger intercept did not significantly deviate from zero and intercept *P*-value > 0.05) did not show significant pleiotropy or heterogeneity (Table 2 and Supplementary Tables 6, 7). In addition, there was no horizontal pleiotropy in MR-PRESSO global test (*P*-value < 0.05). No single SNP significantly affected the estimated causal effects in the LOO analysis (Supplementary Figure 2).

**TABLE 2:** Results of sensitivity analysis for traits that showed significant correlations in both IVW and Weighted median methods.

Exposure	Cochran’s Q	MR-Egger		MR-PRESSO global test	
		Intercept	*P*-value	RSS	*P*-value
CD25++ CD45RA- CD4 not regulatory T cell %T cell	0.646	−2.72E to −02	0.411	4.17	0.65
CD25++ CD45RA- CD4 not regulatory T cell %CD4+ T cell	0.544	−3.79E to −02	0.220	6.52	0.56
Secreting CD4 regulatory T cell %CD4 regulatory T cell	0.879	−2.23E to −03	0.790	7.34	0.88
Activated & secreting CD4 regulatory T cell %CD4 regulatory T cell	0.653	4.22E−03	0.515	12.86	0.67
HLA DR++ monocyte % monocyte	0.289	−2.34E to −03	0.964	NA	NA
HLA DR on myeloid Dendritic Cell	7.92E−05	6.51E−02	0.072	116.00	0.002

IVW, inverse variance weighted; MR-PRESSO, MR pleiotropy residual sum and outlier; RSS, residual sum of squares; NA, not applicable.

### 3.3 Monocyte-related protective traits and AD

As shown in Figure 2 and Table 1, after preliminary analysis, we identified four monocyte-associated immunophenotypes that showed potentially protective effects against AD. HLA DR++ monocyte % monocyte implied a negative association with AD risk in both the IVW analysis method (OR [95% CI] = 0.93 [0.89, 0.98], adjusted *P* = 4.87E−02) and Weighted median analysis (OR [95% CI] = 0.92 [0.88, 0.97], adjusted *P* = 2.40E−02), and the MR-Egger results trended in the same direction (Supplementary Figure 1). HLA DR on the monocyte, CD14+ monocyte, and CD14+ CD16- monocyte showed a potential protective effect against AD only in the IVW analysis (Supplementary Figure 1). The Weighted median analysis results converged in the same direction. Nevertheless, the MR-Egger results hinted that only HLA DR on monocyte tended toward the same direction.

Subsequently, we performed a further sensitivity analysis of HLA DR++ monocyte % monocyte (Table 2). Cochran’s Q test (*Q*-value > 0.05) did not detect heterogeneity (Supplementary Table 6). Moreover, the MR-Egger regression intercept was also insignificant, suggesting an absence of horizontal pleiotropy (Supplementary Table 7). We performed pleiotropy analyses using the PhenoScanner database and found no other relevant traits of instrumental SNPs affecting AD.

### 3.4 Dendritic cell–related and myeloid cell–related protective traits and AD

After screening, based on the IVW approach, we identified two protective immune features associated with dendritic cells and one related to myeloid cells (Figure 2 and Table 1). HLA DR on myeloid Dendritic Cell was dramatically correlated with the IVW method (OR [95% CI] = 0.93 [0.89, 0.97], adjusted *P* = 1.17E−02) and the Weighted median method (OR [95% CI] = 0.92 [0.89, 0.95], adjusted *P* = 8.61E−04); additionally, the MR-Egger results tended to move in the same direction. CD45 on Immature Myeloid-Derived Suppressor Cells and Plasmacytoid Dendritic Cell % Dendritic Cell showed some correlation only in the IVW method.

Subsequently, we conducted an extensive sensitivity analysis of HLA DR on myeloid Dendritic Cell (Table 2). Cochran’s Q test (*Q*-value < 0.001) indicated substantial heterogeneity between instrumental variables, and then a random effects model (IVW) was used to estimate the MR effect size (Supplementary Table 6). The results (*P*-value < 0.05) suggested that causality existed between HLA DR on myeloid Dendritic Cell and AD risk. The sensitivity analysis showed no evidence of heterogeneity or pleiotropy based on the MR-PRESSO global test and the MR-Egger intercept test, and the weighted median analysis was also significant.

### 3.5 Validation analysis

The validation was conducted using AD GWAS data from IGAP. Select independent and significant SNPs according to the same criteria, and perform a two-sample MR analysis. Results indicate that HLA DR on myeloid dendritic cells is statistically significant in the IVW method (OR [95% CI] = 0.89 [0.84, 0.94], adjusted *P* = 7.42E−03) and the Weighted median method (OR [95% CI] = 0.88 [0.83, 0.93], adjusted *P* = 4.72E−04) (Table 3, Supplementary Table 8, and Supplementary Figure 3).

**TABLE 3:** Mendelian randomization (MR) estimates of HLA DR on myeloid dendritic cells on risk of Alzheimer’s disease (AD) using the International Genomics of Alzheimer’s Project (IGAP).

Method	N(SNPs)	Odds ratio (95%CI)	*P*-value (adjusted)
Inverse variance weighted	5	0.89 (0.84, 0.94)	7.42E−03
Weighted median	5	0.88 (0.83, 0.93)	4.72E−04
Simple mode	5	0.90 (0.77, 1.04)	9.60E−01
Weighted mode	5	0.87 (0.82, 0.93)	4.24E−01
MR-Egger	5	0.78 (0.67, 0.92)	9.96E−01

AD, Alzheimer’s disease; IGAP, International Genomics of Alzheimer’s Project; MR, Mendelian randomization; SNP, single nucleotide polymorphism; CI, confidence interval.

## 4 Discussion

Utilizing the largest published GWAS of peripheral immune cell phenotypes to date, we evaluated the causal relationship between peripheral immune cell traits and AD risk by two-sample MR analysis. To data this is the most comprehensive MR study exploring a potential protective causal relationship between peripheral immune cell traits and AD. Mendelian randomization analysis uses genetic variants strongly associated with exposure as instrumental variables to infer causal relationships between exposure and outcome while avoiding bias from various confounding factors and reverse causal associations. In the present study, we identified a total of six peripheral immune phenotypes significantly associated with a low risk of AD, including four Treg cell-associated immune phenotypes (CD25++ CD45RA- CD4 not regulatory T cell % T cell, CD25++ CD45RA- CD4 not regulatory T cell % CD4+ T cell, Secreting CD4 regulatory T cell Absolute Count and Activated & secreting CD4 regulatory T cell Absolute Count), one monocyte-associated phenotype (HLA DR++ monocyte % monocyte), and one belonging to a dendritic cell subpopulation (HLA DR on myeloid Dendritic Cell).

Our research discovered four immune phenotypes linked to Treg cells (CD25++ CD45RA- CD4 not regulatory T cell % T cell, CD25++ CD45RA- CD4 not regulatory T cell % CD4+ T cell, Secreting CD4 regulatory T cell Absolute Count and Activated & secreting CD4 regulatory T cell Absolute Count), suggesting that Treg cells may play a significant protective role in the progression of AD. Interestingly, different activity states of Treg cells were observed. Treg cells can be divided into activated (CD25+++ CD45RA-), resting (CD25++ CD45RA +), and secreting (CD25++CD45RA -) types according to the cell surface markers (Miyara et al., 2009), each exhibiting distinct functions. Our findings indicate that the Treg cell immunophenotypes contributing to protection in AD are predominantly characterized by the secreting and activated types. Activated Treg cells are generated largely from resting Tregs after exposure to self-antigens and express high levels of CD25 (Lykhopiy et al., 2023). CD25, encoded by the gene interleukin (IL)2 receptor alpha, is the α-chain of the IL-2 receptor, a component of the receptor complex. It mediates the effects of IL-2 and efficiently uses IL-2 to promote the survival and proliferation of Treg cells, thereby maintaining an anti-inflammatory environment in the immune system. Biological evidence from an experimental AD mouse model has explained that IL-2 triggers the activation of Tregs and astrocytes in APP/PS1 mice and increases the recruitment of astrocytes around amyloid plaques, reducing Aβ and slowing the development of AD (Alves et al., 2016). Secreting Treg cells can release a large number of cytokines such as IL-10 and transforming growth factor-beta (TGF-β) to suppress inflammatory responses, thereby protecting neurons from damage (Sanjabi et al., 2009; Saraiva and O’Garra, 2010; Kapoor and Chinnathambi, 2023). Research indicates that reduced production of anti-inflammatory TGF-β heightens the risk of developing AD in individuals with mild cognitive impairment (Tarkowski, 2003). Caraci et al. (2011) found that compared to healthy elderly individuals, AD patients have lower levels of TGF-β1 in their plasma and serum, and a reduced release of TGF-β1 by circulating peripheral blood cells (Caraci et al., 2011).

Monocytes play a key role in the pathogenesis of AD through immune regulation, inflammatory responses, and the elimination of Aβ. Our research demonstrates a significant negative correlation between the immunophenotype HLA DR++ monocyte % monocyte and AD risk. HLA DR is a major histocompatibility complex (MHC) class II molecule, primarily involved in antigen presentation in the immune system. Enhanced expression of HLA DR++ indicates increased antigen-presenting capabilities of monocytes. In the progression of AD, monocytes might facilitate more efficient clearance of Aβ. A recent study reported that in an APP/PS1/Cx3cr1 AD mouse model, patrolling monocytes could climb up the lumen wall of Aβ-positive veins and target Aβ clearance from the venous lumen, as observed by in-vivo two-photon microscopy (Michaud et al., 2013). The selective removal of these monocytes resulted in a significant increase in Aβ load in the brain of the APP/PS1 mice. A recent MR study found an inverse association between monocyte count and AD risk, and our MR analysis obtained consistent results (Luo et al., 2022).

MR analysis indicates that HLA DR on myeloid Dendritic Cell is significantly associated with a reduced risk of AD, with similar results obtained in the validation cohort. Dendritic cells (DCs) can be divided into plasma cell-derived (CD123+) and myeloid-derived (CD11c+) according to their origin (Ziegler-Heitbrock et al., 2010). Myeloid DCs (mDCs) are professional antigen-presenting cells that present antigens to T cells via HLA DR molecules, activating a specific immune response (Banchereau et al., 2000). In AD, higher HLA DR expression in mDCs may enhance T-cell mediated immune clearance against Aβ. This partially explains why high expression of HLA DR in mDCs can reduce the risk of developing AD. Furthermore, research conducted by Ciaramella et al. (2016) showed that in the peripheral blood of AD patients, the number of mDCs was specifically reduced compared to healthy controls while the plasma DC (pDC) subpopulation remained unchanged, this suggests that the reduction in blood mDCs may be related to the progression of AD. Our findings of a negative association between peripheral mDCs with the high expression of HLA DR and AD risk likely reinforce the innovative idea that blood mDC represents a potential participant in AD from an epidemiological perspective.

Although in the validation cohort, we only observed a significant protective effect of HLA DR on myeloid Dendritic Cell on AD, we believe that this may be related to the following factors. Firstly, there is a significant disparity in the sample sizes of the two AD cohorts. Compared to IGAP’s GWAS summary data, the EADB cohort offers stronger statistical power, facilitating the detection of subtle associations. Secondly, Moreover, genetic diversity and stratification across populations could result in variations in genetic risk expression. Furthermore, the EADB dataset comprises approximately 21 million SNPs, in contrast to about 7 million SNPs in the IGAP dataset. Variations in SNP coverage and genotyping approaches between the datasets might impact the intensity and identification of genetic associations.

The limitations of our study are as follows: (1) the GWAS data on exposure used in this study were derived from the Sardinian population only, and although the Mediterranean Sardinian population has been exhaustively used for genetic analysis, some of the immune traits and associations reported may be driven by genetic variants that are more common in the Sardinian population than elsewhere, and GWAS data on peripheral blood immune phenotypes from other ethnic groups may be needed to validate the results further. (2) The sample size of GWAS data for exposure and the number of SNPs obtained was comparatively tiny. Future studies using more extensive GWAS databases for immune cell traits are needed. However, in our study, the F-statistic value was used as the condition to measure the strength of instrumental variables, and only the instrumental variables with *F* > 10 were used in the subsequent analysis. Therefore, our findings were considered reliable. (3) The results based on the GWAS data of European ancestry may not apply to other ethnic populations and require further validation by GWAS data of other ethnic groups.

## 5 Conclusion

In summary, we primarily identified several immunophenotypes in Tregs, monocytes, and mDCs that were associated with an appropriate reduction in AD risk. Our work further validated the idea that peripheral immune disorders play an important role in the progression of AD, and these immunophenotypes may become potential biomarkers for predicting disease progression, providing new insights into potential immunotherapy targets for AD.

## Data availability statement

The original contributions presented in this study are included in this article/Supplementary material, further inquiries can be directed to the corresponding author.

## Author contributions

C-yZ: Writing – original draft, Visualization, Validation, Software, Methodology, Data curation. ZH: Writing – original draft, Visualization, Validation, Software, Data curation. X-yH: Writing – original draft, Visualization. M-jL: Writing – original draft, Visualization. J-jS: Writing – original draft, Validation, Data curation. M-nG: Writing – original draft, Formal Analysis, Data curation. D-rM: Writing – original draft, Validation, Software, Methodology. S-jL: Writing – original draft, Methodology, Data curation. Y-yL: Writing – original draft, Visualization, Validation. CZ: Writing – review and editing, Visualization, Software. C-yM: Writing – review and editing, Visualization, Validation, Data curation. YX: Writing – review and editing, Data curation. C-hS: Writing – review and editing, Visualization, Validation, Software, Data curation.

## Footnotes

1
https://shiny.cnsgenomics.com/mRnd/

## References

[B1] Alves S. Churlaud G. Audrain M. Michaelsen-Preusse K. Fol R. Souchet B. (2016). Interleukin-2 improves amyloid pathology, synaptic failure and memory in Alzheimer’s disease mice. *Brain* 140 826–842. 10.1093/brain/aww330 28003243

[B2] Aries M. L. Hensley-Mcbain T. (2023). Neutrophils as a potential therapeutic target in Alzheimer’s disease. *Front. Immunol.* 14:1123149. 10.3389/fimmu.2023.1123149 36936930 PMC10020508

[B3] Banchereau J. Briere F. Caux C. Davoust J. Lebecque S. Liu Y.-J. (2000). Immunobiology of dendritic cells. *Annu. Rev. Immunol.* 18 767–811. 10.1146/annurev.immunol.18.1.767 10837075

[B4] Bellenguez C. Küçükali F. Jansen I. E. Kleineidam L. Moreno-Grau S. Amin N. (2022). New insights into the genetic etiology of Alzheimer’s disease and related dementias. *Nat. Genet.* 54 412–436. 10.1038/s41588-022-01024-z 35379992 PMC9005347

[B5] Bowden J. Davey Smith G. Burgess S. (2015). Mendelian randomization with invalid instruments: Effect estimation and bias detection through Egger regression. *Int. J. Epidemiol.* 44 512–525. 10.1093/ije/dyv080 26050253 PMC4469799

[B6] Bowden J. Davey Smith G. Haycock P. C. Burgess S. (2016). Consistent estimation in Mendelian randomization with some invalid instruments using a weighted median estimator. *Genet. Epidemiol.* 40 304–314. 10.1002/gepi.21965 27061298 PMC4849733

[B7] Bowden J. Del Greco M. F. Minelli C. Zhao Q. Lawlor D. A. Sheehan N. A. (2019). Improving the accuracy of two-sample summary-data Mendelian randomization: Moving beyond the NOME assumption. *Int. J. Epidemiol.* 48 728–742. 10.1093/ije/dyy258 30561657 PMC6659376

[B8] Brion M.-J. A. Shakhbazov K. Visscher P. M. (2013). Calculating statistical power in Mendelian randomization studies. *Int. J. Epidemiol.* 42 1497–1501. 10.1093/ije/dyt179 24159078 PMC3807619

[B9] Burgess S. (2014). Sample size and power calculations in Mendelian randomization with a single instrumental variable and a binary outcome. *Int. J. Epidemiol.* 43 922–929. 10.1093/ije/dyu005 24608958 PMC4052137

[B10] Burgess S. Thompson S. G. (2011). Avoiding bias from weak instruments in Mendelian randomization studies. *Int. J. Epidemiol.* 40 755–764. 10.1093/ije/dyr036 21414999

[B11] Burgess S. Butterworth A. Malarstig A. Thompson S. G. (2012). Use of Mendelian randomisation to assess potential benefit of clinical intervention. *BMJ* 345 e7325–e7325. 10.1136/bmj.e7325 23131671

[B12] Caraci F. Battaglia G. Bruno V. Bosco P. Carbonaro V. Giuffrida M. L. (2011). TGF-β1 pathway as a new target for neuroprotection in Alzheimer’s disease. *CNS Neurosci. Ther.* 17 237–249. 10.1111/j.1755-5949.2009.00115.x 19925479 PMC6493850

[B13] Carson M. J. Doose J. M. Melchior B. Schmid C. D. Ploix C. C. (2006). CNS immune privilege: Hiding in plain sight. *Immunol. Rev.* 213 48–65. 10.1111/j.1600-065x.2006.00441.x 16972896 PMC2633103

[B14] Ciaramella A. Salani F. Bizzoni F. Orfei M. D. Caltagirone C. Spalletta G. (2016). Myeloid dendritic cells are decreased in peripheral blood of Alzheimer’s disease patients in association with disease progression and severity of depressive symptoms. *J. Neuroinflamm.* 13:18. 10.1186/s12974-016-0483-0 26811068 PMC4727381

[B15] Davey Smith G. Ebrahim S. (2003). ‘Mendelian randomization’: Can genetic epidemiology contribute to understanding environmental determinants of disease?*. *Int. J. Epidemiol.* 32 1–22. 10.1093/ije/dyg070 12689998

[B16] Desai B. S. Monahan A. J. Carvey P. M. Hendey B. (2007). Blood–brain barrier pathology in Alzheimer’s and Parkinson’s disease: Implications for drug therapy. *Cell Transplant.* 16 285–299. 10.3727/000000007783464731 17503739

[B17] Engelhardt B. Ransohoff R. M. (2005). The ins and outs of T-lymphocyte trafficking to the CNS: Anatomical sites and molecular mechanisms. *Trends Immunol.* 26 485–495. 10.1016/j.it.2005.07.004 16039904

[B18] Glymour M. M. Tchetgen E. J. Robins J. M. (2012). Credible Mendelian randomization studies: Approaches for evaluating the instrumental variable assumptions. *Am. J. Epidemiol.* 175 332–339. 10.1093/aje/kwr323 22247045 PMC3366596

[B19] Hansen D. V. Hanson J. E. Sheng M. (2018). Microglia in Alzheimer’s disease. *J. Cell Biol.* 217 459–472. 10.1083/jcb.201709069 29196460 PMC5800817

[B20] Hartwig F. P. Davies N. M. Hemani G. Davey Smith G. (2016). Two-sample Mendelian randomization: Avoiding the downsides of a powerful, widely applicable but potentially fallible technique. *Int. J. Epidemiol.* 45 1717–1726. 10.1093/ije/dyx028 28338968 PMC5722032

[B21] Hemani G. Zheng J. Elsworth B. Wade K. H. Haberland V. Baird D. (2018). The MR-base platform supports systematic causal inference across the human phenome. *Elife* 7:e34408. 10.7554/elife.34408 29846171 PMC5976434

[B22] Hemonnot A.-L. Hua J. Ulmann L. Hirbec H. (2019). Microglia in Alzheimer disease: Well-known targets and new opportunities. *Front. Aging Neurosci.* 11:233. 10.3389/fnagi.2019.00233 31543810 PMC6730262

[B23] Kamat M. A. Blackshaw J. A. Young R. Surendran P. Burgess S. Danesh J. (2019). PhenoScanner V2: An expanded tool for searching human genotype–phenotype associations. *Bioinformatics* 35 4851–4853. 10.1093/bioinformatics/btz469 31233103 PMC6853652

[B24] Kapoor M. Chinnathambi S. (2023). TGF-β1 signalling in Alzheimer’s pathology and cytoskeletal reorganization: A specialized Tau perspective. *J. Neuroinflamm.* 20:72. 10.1186/s12974-023-02751-8 36915196 PMC10012507

[B25] Lambert J.-C. Ibrahim-Verbaas C. A. Harold D. Naj A. C. Sims R. Bellenguez C. (2013). Meta-analysis of 74,046 individuals identifies 11 new susceptibility loci for Alzheimer’s disease. *Nat. Genet.* 45 1452–1458. 10.1038/ng.2802 24162737 PMC3896259

[B26] Lawlor D. A. Harbord R. M. Sterne J. A. C. Timpson N. Davey Smith G. (2008). Mendelian randomization: Using genes as instruments for making causal inferences in epidemiology. *Stat. Med.* 27 1133–1163. 10.1002/sim.3034 17886233

[B27] Lueg G. Gross C. C. Lohmann H. Johnen A. Kemmling A. Deppe M. (2015). Clinical relevance of specific T-cell activation in the blood and cerebrospinal fluid of patients with mild Alzheimer’s disease. *Neurobiol. Aging* 36 81–89. 10.1016/j.neurobiolaging.2014.08.008 25277040

[B28] Luo J. Thomassen J. Q. Nordestgaard B. G. Tybjærg-Hansen A. Frikke-Schmidt R. (2022). Blood leukocyte counts in Alzheimer disease. *JAMA Netw. Open* 5:e2235648. 10.1001/jamanetworkopen.2022.35648 36215071 PMC9552891

[B29] Lykhopiy V. Malviya V. Humblet-Baron S. Schlenner S. M. (2023). IL-2 immunotherapy for targeting regulatory T cells in autoimmunity. *Genes Immun.* 24 248–262. 10.1038/s41435-023-00221-y 37741949 PMC10575774

[B30] Michaud J.-P. Bellavance M.-A. Préfontaine P. Rivest S. (2013). Real-time in vivo imaging reveals the ability of monocytes to clear vascular amyloid beta. *Cell Rep.* 5 646–653. 10.1016/j.celrep.2013.10.010 24210819

[B31] Miyara M. Yoshioka Y. Kitoh A. Shima T. Wing K. Niwa A. (2009). Functional delineation and differentiation dynamics of human CD4+ T cells expressing the FoxP3 transcription factor. *Immunity* 30 899–911. 10.1016/j.immuni.2009.03.019 19464196

[B32] Orrù V. Steri M. Sidore C. Marongiu M. Serra V. Olla S. (2020). Complex genetic signatures in immune cells underlie autoimmunity and inform therapy. *Nat. Genet.* 52 1036–1045. 10.1038/s41588-020-0684-4 32929287 PMC8517961

[B33] Palmer T. M. Sterne J. A. C. Harbord R. M. Lawlor D. A. Sheehan N. A. Meng S. (2011). Instrumental variable estimation of causal risk ratios and causal odds ratios in Mendelian randomization analyses. *Am. J. Epidemiol.* 173 1392–1403. 10.1093/aje/kwr026 21555716

[B34] Pierce B. L. Ahsan H. Vanderweele T. J. (2011). Power and instrument strength requirements for Mendelian randomization studies using multiple genetic variants. *Int. J. Epidemiol.* 40 740–752. 10.1093/ije/dyq151 20813862 PMC3147064

[B35] Reitz C. Mayeux R. (2014). Alzheimer disease: Epidemiology, diagnostic criteria, risk factors and biomarkers. *Biochem. Pharmacol.* 88 640–651. 10.1016/j.bcp.2013.12.024 24398425 PMC3992261

[B36] Sanjabi S. Zenewicz L. A. Kamanaka M. Flavell R. A. (2009). Anti-inflammatory and pro-inflammatory roles of TGF-β, IL-10, and IL-22 in immunity and autoimmunity. *Curr. Opin. Pharmacol.* 9 447–453. 10.1016/j.coph.2009.04.008 19481975 PMC2755239

[B37] Saraiva M. O’Garra A. (2010). The regulation of IL-10 production by immune cells. *Nat. Rev. Immunol.* 10 170–181. 10.1038/nri2711 20154735

[B38] Serrano-Pozo A. Frosch M. P. Masliah E. Hyman B. T. (2011). Neuropathological alterations in Alzheimer disease. *Cold Spring Harbor Perspect. Med.* 1:a006189. 10.1101/cshperspect.a006189 22229116 PMC3234452

[B39] Sevigny J. Chiao P. Bussière T. Weinreb P. H. Williams L. Maier M. (2016). The antibody aducanumab reduces Aβ plaques in Alzheimer’s disease. *Nature* 537 50–56. 10.1038/nature19323 27582220

[B40] Skias D. Bania M. Reder A. T. Luchins D. Antel J. P. (1985). Senile dementia of Alzheimer’s type (SDAT). *Neurology* 35 1635–1635. 10.1212/wnl.35.11.1635 2932656

[B41] Skrivankova V. W. Richmond R. C. Woolf B. A. R. Yarmolinsky J. Davies N. M. Swanson S. A. (2021). Strengthening the reporting of observational studies in epidemiology using Mendelian randomization. *JAMA* 326:1614. 10.1001/jama.2021.18236 34698778

[B42] Sperling R. Salloway S. Brooks D. J. Tampieri D. Barakos J. Fox N. C. (2012). Amyloid-related imaging abnormalities in patients with Alzheimer’s disease treated with bapineuzumab: A retrospective analysis. *Lancet Neurol.* 11 241–249. 10.1016/s1474-4422(12)70015-7 22305802 PMC4063417

[B43] Tarkowski E. (2003). Intrathecal inflammation precedes development of Alzheimer’s disease. *J. Neurol. Neurosurg. Psychiatry* 74 1200–1205. 10.1136/jnnp.74.9.1200 12933918 PMC1738668

[B44] Verbanck M. Chen C.-Y. Neale B. Do R. (2018). Detection of widespread horizontal pleiotropy in causal relationships inferred from Mendelian randomization between complex traits and diseases. *Nat. Genet.* 50 693–698. 10.1038/s41588-018-0099-7 29686387 PMC6083837

[B45] Wilcock D. M. Rojiani A. Rosenthal A. Subbarao S. Freeman M. J. Gordon M. N. (2004). Passive immunotherapy against Abeta in aged APP-transgenic mice reverses cognitive deficits and depletes parenchymal amyloid deposits in spite of increased vascular amyloid and microhemorrhage. *J. Neuroinflamm.* 1:24. 10.1186/1742-2094-1-24 15588287 PMC539292

[B46] Woolf B. Di Cara N. Moreno-Stokoe C. Skrivankova V. Drax K. Higgins J. P. T. (2022). Investigating the transparency of reporting in two-sample summary data Mendelian randomization studies using the MR-base platform. *Int. J. Epidemiol.* 51 1943–1956. 10.1093/ije/dyac074 35383846 PMC9749715

[B47] Xu H. Jia J. (2021). Single-Cell RNA sequencing of peripheral blood reveals immune cell signatures in Alzheimer’s disease. *Front. Immunol.* 12:645666. 10.3389/fimmu.2021.645666 34447367 PMC8382575

[B48] Ziegler-Heitbrock L. (2007). The CD14+ CD16+ blood monocytes: Their role in infection and inflammation. *J. Leukocyte Biol.* 81 584–592. 10.1189/jlb.0806510 17135573

[B49] Ziegler-Heitbrock L. Ancuta P. Crowe S. Dalod M. Grau V. Hart D. N. (2010). Nomenclature of monocytes and dendritic cells in blood. *Blood* 116 e74–e80. 10.1182/blood-2010-02-258558 20628149

