# Supplementary material for: The potential protective role of peripheral immunophenotypes in Alzheimer’s disease: a Mendelian randomization study
Source: Front Aging Neurosci. 2024 Jun 5;16:1403077. doi: 10.3389/fnagi.2024.1403077 (PMC11188398; doi:10.3389/fnagi.2024.1403077)
Supplement: Supplementary file 2 [file Data_Sheet_1.docx]

Supplementary Material

***Supplementary Tables Legends:***

**Supplementary Table 1:** The detailed characteristics of the instrumental SNPs associated with 731 immune traits used in this study derived from European Alzheimer’s and Dementia Biobank (EADB).

**Supplementary Table 2:** MR results of all immune traits identified in different MR methods derived from European Alzheimer’s and Dementia Biobank (EADB).

**Supplementary Table 3:** FDR-adjusted MR results of all immune traits identified in IVW method derived from European Alzheimer’s and Dementia Biobank (EADB).

**Supplementary Table 4:** FDR-adjusted MR results of all immune traits identified in Weighted median method using the European Alzheimer’s and Dementia Biobank (EADB).

**Supplementary Table 5:** FDR-adjusted MR results of all immune traits identified in MR-Egger method using the European Alzheimer’s and Dementia Biobank (EADB).

**Supplementary Table 6:**Results of heterogeneity analysis of 6 protective immune traits using the European Alzheimer’s and Dementia Biobank (EADB) that showed significant positive results by both IVW and Weighted Median methods.

**Supplementary Table 7:** Results of pleiotropy analysis of 6 protective immune traits that showed significant positive results by both IVW and Weighted median methods derived from European Alzheimer’s and Dementia Biobank (EADB).

**Supplementary Table 8:** MR results of all immune traits identified in different MR methods using the International Genomics of Alzheimer’s Project (IGAP).

**Supplementary Table 9:** Specific SNPS found in Mendelian randomization analysis and their mapping genes.

***Supplementary Figures Legends:***

**Supplementary Figure 1:** Scatter plot for the relationship between the SNP effect size of causal immune traits and the corresponding effect size estimates of AD. (A) CD25++ CD45RA- CD4 not regulatory T cell %T cell, (B) CD25++ CD45RA- CD4 not regulatory T cell %CD4+ T cell, (C) Secreting CD4 regulatory T cell %CD4 regulatory T cell, (D) Activated & secreting CD4 regulatory T cell %CD4 regulatory T cell, (E) HLA DR++ monocyte %monocyte, (F) HLA DR on myeloid Dendritic Cell. AD, Alzheimer’s disease.

**Supplementary Figure 2:** Leave-one-out plot for single SNP effect size of causal immune traits on AD. (A) CD25++ CD45RA- CD4 not regulatory T cell %T cell, (B) CD25++ CD45RA- CD4 not regulatory T cell %CD4+ T cell, (C) Secreting CD4 regulatory T cell %CD4 regulatory T cell, (D) Activated & secreting CD4 regulatory T cell %CD4 regulatory T cell, (E) HLA DR++ monocyte %monocyte, (F) HLA DR on myeloid Dendritic Cell. AD, Alzheimer’s disease.

**Supplementary Figure 3:** Visualization results of sensitivity analysis of HLA DR on myeloid Dendritic cells. (A) Forest plot for the SNP effect size of HLA DR on myeloid Dendritic cells and the corresponding effect size estimates of AD. (B) Leave-one-out plot for the SNP effect size of HLA DR on myeloid Dendritic cells and the corresponding effect size estimates of AD. (C) Scatter plot for the relationship between the SNP effect size of HLA DR on myeloid Dendritic cells and the corresponding effect size estimates of AD. (D) Funnel plot for the relationship between the SNP effect size of HLA DR on myeloid Dendritic cells and the corresponding effect size estimates of AD. AD, Alzheimer’s disease.
