# Supplementary figures and images for: The potential protective role of peripheral immunophenotypes in Alzheimer’s disease: a Mendelian randomization study
Source: Front Aging Neurosci. 2024 Jun 5;16:1403077. doi: 10.3389/fnagi.2024.1403077 (PMC11188398; doi:10.3389/fnagi.2024.1403077)

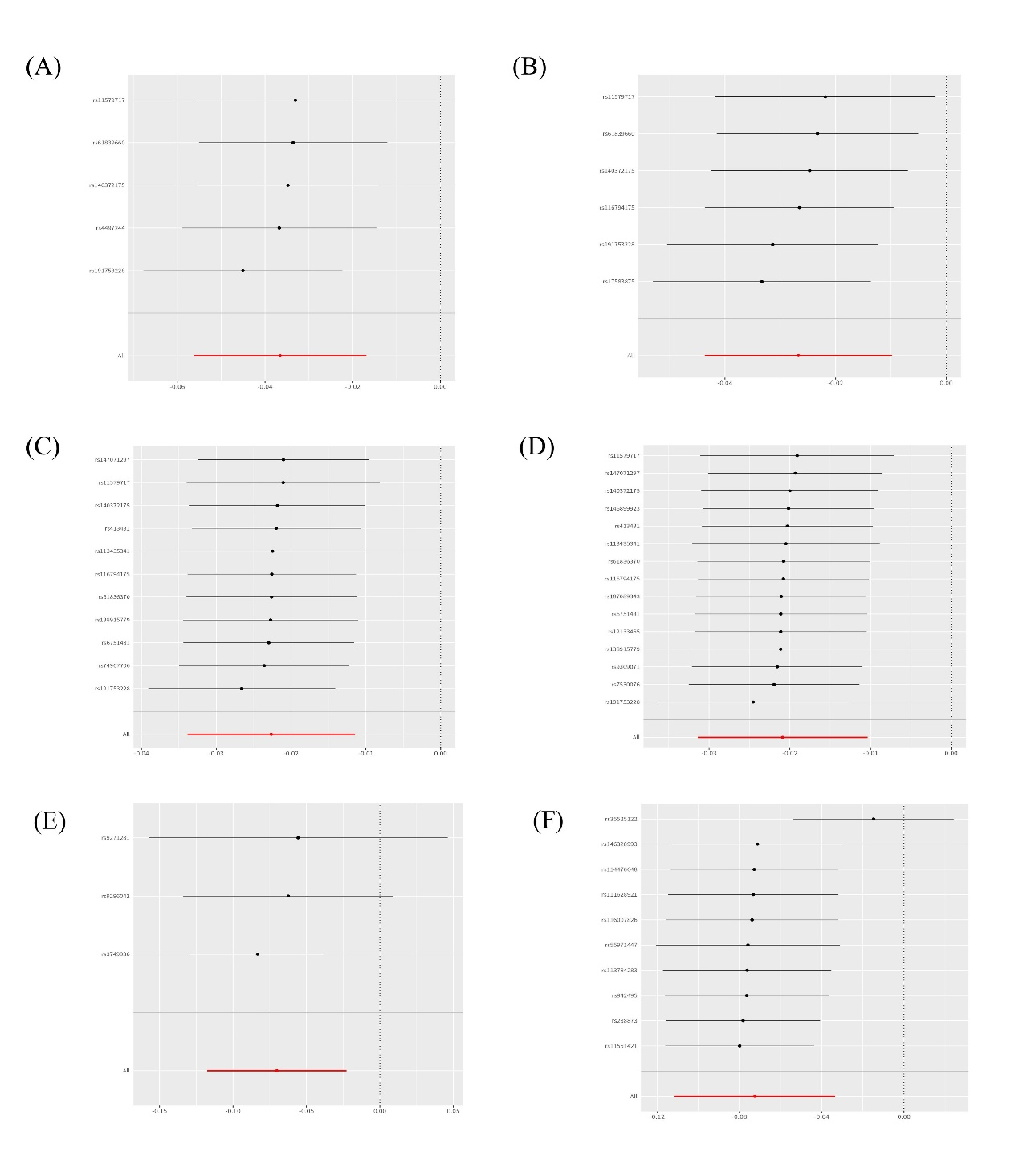

Supplement: Supplementary file 3 [file Image_1.TIF]

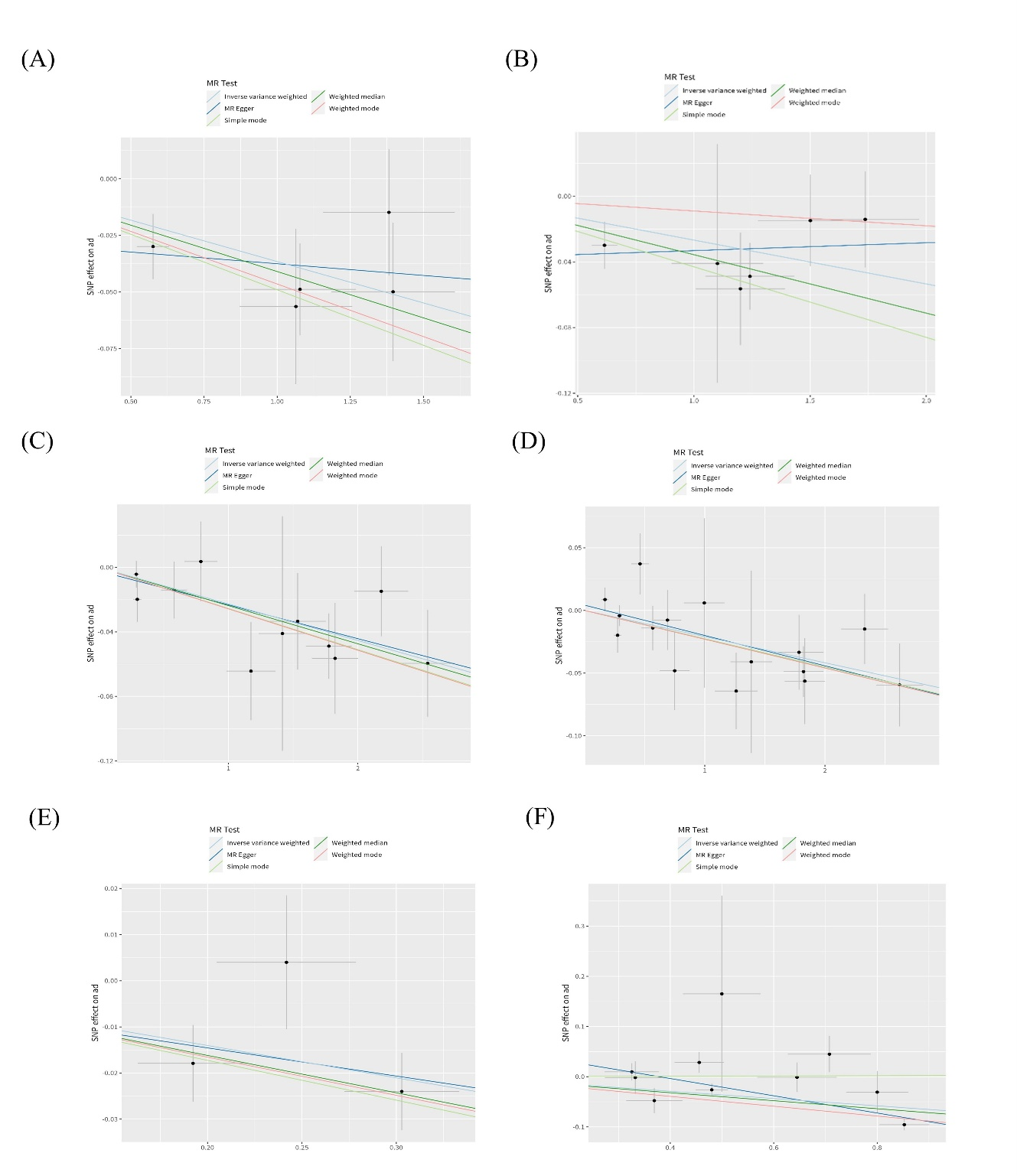

Supplement: Supplementary file 4 [file Image_2.TIF]

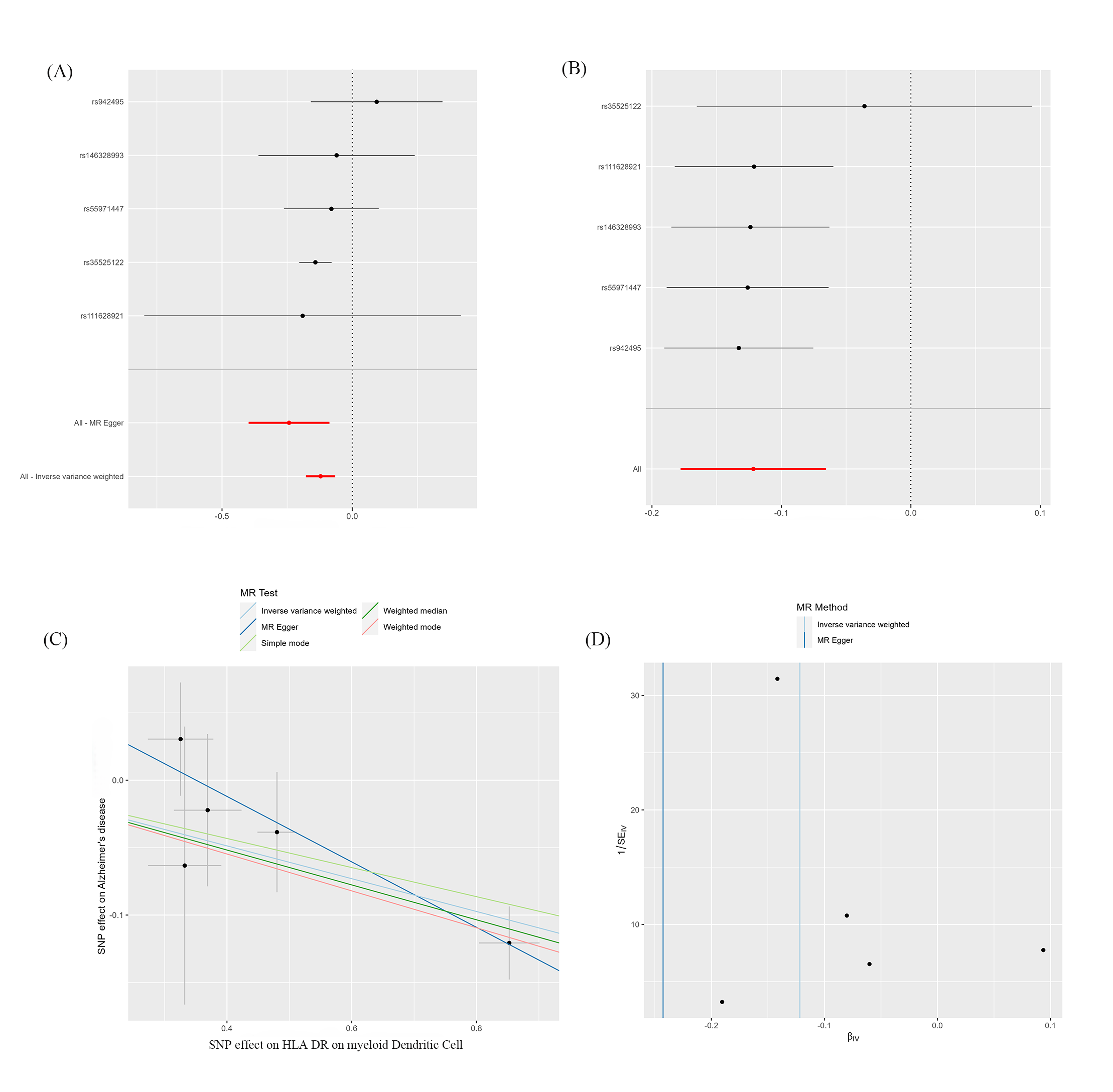

Supplement: Supplementary file 5 [file Image_3.TIF]
